# Does Environmental Enrichment Reduce Stress? An Integrated Measure of Corticosterone from Feathers Provides a Novel Perspective

**DOI:** 10.1371/journal.pone.0017663

**Published:** 2011-03-11

**Authors:** Graham D. Fairhurst, Matthew D. Frey, James F. Reichert, Izabela Szelest, Debbie M. Kelly, Gary R. Bortolotti

**Affiliations:** 1 Department of Biology, University of Saskatchewan, Saskatoon, Saskatchewan, Canada; 2 Department of Psychology, University of Saskatchewan, Saskatoon, Saskatchewan, Canada; Semmelweis University, Hungary

## Abstract

Enrichment is widely used as tool for managing fearfulness, undesirable behaviors, and stress in captive animals, and for studying exploration and personality. Inconsistencies in previous studies of physiological and behavioral responses to enrichment led us to hypothesize that enrichment and its removal are stressful environmental changes to which the hormone corticosterone and fearfulness, activity, and exploration behaviors ought to be sensitive. We conducted two experiments with a captive population of wild-caught Clark's nutcrackers (*Nucifraga columbiana*) to assess responses to short- (10-d) and long-term (3-mo) enrichment, their removal, and the influence of novelty, within the same animal. Variation in an integrated measure of corticosterone from feathers, combined with video recordings of behaviors, suggests that how individuals perceive enrichment and its removal depends on the duration of exposure. Short- and long-term enrichment elicited different physiological responses, with the former acting as a stressor and birds exhibiting acclimation to the latter. Non-novel enrichment evoked the strongest corticosterone responses of all the treatments, suggesting that the second exposure to the same objects acted as a physiological cue, and that acclimation was overridden by negative past experience. Birds showed weak behavioral responses that were not related to corticosterone. By demonstrating that an integrated measure of glucocorticoid physiology varies significantly with changes to enrichment in the absence of agonistic interactions, our study sheds light on potential mechanisms driving physiological and behavioral responses to environmental change.

## Introduction

Enrichment is the modification of a captive animal's environment with the goals of increasing environmental complexity [1] and improving biological functioning [2]. The majority of enrichment research has focused on combating fearfulness and harmful abnormal and stereotypic behaviors arising in captive production (i.e., farm), laboratory, and companion animals ([3]; see also [4]) because animal welfare is both economically and ethically important [1], [5]–[7]. Behavioral ecologists have indirectly studied enrichment in different contexts, and more frequently use non-domesticated animals as models. Investigations of, for example, exploration behavior [8], [9] information acquisition [10], dominance [11], and personality [12], [13] can involve *de facto* enrichment and provide data comparable to studies of other captive animals. Enrichment has numerous behavioral effects (for reviews see [6], [14], [15]), but has been shown to reduce fear responses [1], [13], [16], increase movement and activity [17]–[19], and induce changes in exploration behavior [8], [10], [11], [20], [21].

Studies assessing physiological responses to enrichment frequently measure levels of glucocorticoid (GC) hormones like corticosterone (CORT) or cortisol because they vary with exposure to environmental perturbations [22]–[25]. Prolonged activation of the HPA axis and sustained elevated levels of GCs have detrimental effects on health and reproduction [26], [27] and GC levels can correlate with fitness components [25], [28]–[30]. However, while some studies have reported that enrichment can lower GC levels in the blood [31], [32], others have reported no effect [19], [20], [33], [34], or even increases [21], [35], [36]. Such studies have been inconsistent in their procedures for measuring GC physiology and there is still a lack of consensus and understanding of the effects of enrichment on GCs [37], [38]. Furthermore, studies addressing simultaneous behavioral and physiological responses to enrichment have found that animals may react behaviorally in measurable ways yet exhibit no measurable GC response. For example, garden warblers (*Sylvia borin*) exposed to a toy exhibited active exploration [20] and great tits (*Parus major*) exposed to a box showed increased activity [19], yet neither showed a change in circulating CORT levels. Likewise, steers (*Bos primigenius*) given a drum can [39] and calves given toys [40] significantly increased active behaviors, yet showed no change in cortisol levels. How abnormal behaviors relate to stressors is unclear. For example, Dybkjaer [41] reported that belly-nosing behavior in pigs is an indicator of stressful rearing conditions, but when Gardner and colleagues [42] manipulated pig density as a means of lowering stress, they did not detect a change in that behavior. Furthermore, Le Maho and colleagues [43] found that although domestic geese appeared calm and exhibited no behavioral signs of stress during a routine procedure to which they had been adjusted, several-fold increases in CORT levels were detected following the procedure. This collective evidence suggests that behavior and stress physiology are context dependent and may operate independently of each other.

From the perspective of the animal, enrichment constitutes an unpredictable environmental change. Thus, an animal's response to enrichment may not be caused by the enrichment objects *per se*, but rather by the associated change. Although some behavioral responses to enrichment, such as exploration and play, can be attributed to the objects themselves, physiological responses may more likely be caused by the unpredictable nature of the change in environment. Vertebrates are well known to respond physiologically to such change by releasing GCs as part of the “stress response” [23], [24], [28].

While the vast majority of research has addressed the effects of enrichment, relatively little is known about how animals respond to a change from an enriched to a more impoverished environment. This is an important knowledge gap because it is an animal's response to removal of enrichment objects that would shed light on the importance of associated environmental change. A barren environment can affect behavior [44] and physiology [45], and sparse evidence suggests that removal of enrichment can have negative physiological [31], [39] and psychological [46] effects. However, studies experimentally testing the relationship between behavioral and GC responses to enrichment and its removal within the same animal are rare, especially for non-domesticated birds [3].

It is important to determine how well a change in behavior correlates with measures of physiological stress [47], especially if behavioral responses to enrichment are to be integrated effectively into measures of emotional state and, subsequently, well-being and quality of life of captive animals (see [7], [46], [48]). Enrichment is used as a stress-reduction technique ([49] and references therein; [50]) and stress is believed to mediate the relationship between problem behaviors and well-being [51]–[53]. However, inconsistencies in the literature make it necessary to clarify how and when the HPA axis responds to enrichment. Better understanding the relationships between enrichment, behavior, and stress will help refine techniques for assessing the outcomes of enrichment procedures [53], which will benefit a broad spectrum of research.

Although the lack of consensus regarding enrichment and GC levels may be partly context dependent (e.g., different enrichment protocols; [37]), all previous studies measuring GC levels have utilized blood or, less frequently, fecal [54]–[56] or salivary [57] sampling. These techniques have known limitations and biases ([58], [59]; and see [60]) and provide measures of GC physiology over short time periods (i.e., minutes or hours). Thus, our understanding of how enrichment affects stress physiology would benefit from a long-term perspective on GC secretion. Here we use a technique to track stress physiology of birds through changes in CORT found in feathers. Feather CORT integrates the intensity and frequency of the physiological response because values incorporate the amplitude and duration of all CORT secretion, including response to stressors, during the period of feather growth [60], [61]. Therefore, feather CORT does not rely solely on baseline or stress-induced values, but instead integrates the two into a biologically-relevant measure of total CORT secretion (*sensu* [24]).

We conducted two experiments to help clarify the relationships between enrichment and its removal, GC physiology, and behavior. Although domestic chickens (*Gallus gallus*) are the typical avian model for enrichment research, we wanted the results of our study to also be applicable to behavioral ecologists, so we used a captive population of wild-caught Clark's nutcrackers (*Nucifraga columbiana*). During experiment 1 we exposed nutcrackers to short-term (10-d) enrichment to test the hypothesis that enrichment attenuates stress physiology. If this were true, nutcracker feather CORT should be significantly reduced following short-term enrichment. Alternatively, if short-term enrichment does not affect nutcracker stress physiology, or if nutcrackers perceive enrichment as a stressor, we predict no effect or an increase in feather CORT, respectively. In experiment 2 we exposed nutcrackers to enrichment objects continuously for three months, then removed the objects. This design allowed us to replicate experiment 1, using both short- and long-term enrichment, and also test the hypothesis that the change of environment associated with enrichment and its removal is perceived as a stressor. If the environmental change were a stressor, feather CORT should increase immediately following both addition and removal of enrichment objects. Additionally, we were interested in how well behavioral measures can be used as a proxy for physiological responses to enrichment, so in experiment 2 we examined the relationships between feather CORT and fearfulness, activity, and exploration behaviors.

## Materials and Methods

### Ethics Statement

All aspects of this research complied fully with the rules and regulations governing the use and care of animals in research at the University of Saskatchewan, and were conducted under approval #20040088 from the Animal Research Ethics Board, University of Saskatchewan.

### Housing and daily routine

During 2000–2002, 41 wild nutcrackers were caught in Colorado, USA, so all birds had been in captivity for at least 4 years prior to our first experiment in 2007. All birds were housed individually at the Western College of Veterinary Medicine, University of Saskatchewan, Canada, in a single windowless colony room in standard metal pet bird cages constructed from thin (∼3 mm) metal bars with a removable metal floor tray (1 m×0.75 m×1 m). All cages had a wooden perch and separate wood and metal swing. All birds were checked regularly by veterinarians and were deemed in good health before we began our experiments. Prior to experiments, all birds had experienced the same daily cleaning and feeding routine that we continued for the duration of the experiments: morning weighing, feeding, and water changing; afternoon water changing; weekly cage changes; and additional twice weekly cage bottom cleaning. Nutcrackers were fed a 95% *ad libitum* diet of turkey starter, parrot pellets, sunflower seeds, peanuts, pine nuts, mealworms, and vitamin supplement, as well as water and grit *ad libitum*. Food and grit was provided in plastic food cups snapped into the cage walls, and water was provided in circular plastic bowls. Cages were arranged on moveable racks that could accommodate three cages above and three cages below. Light was maintained at 12 h light:12 h dark. None of the birds in our experiments had previously received any form of cage enrichment other than their perch and swing.

### Experiment 1

**Figure 1:**
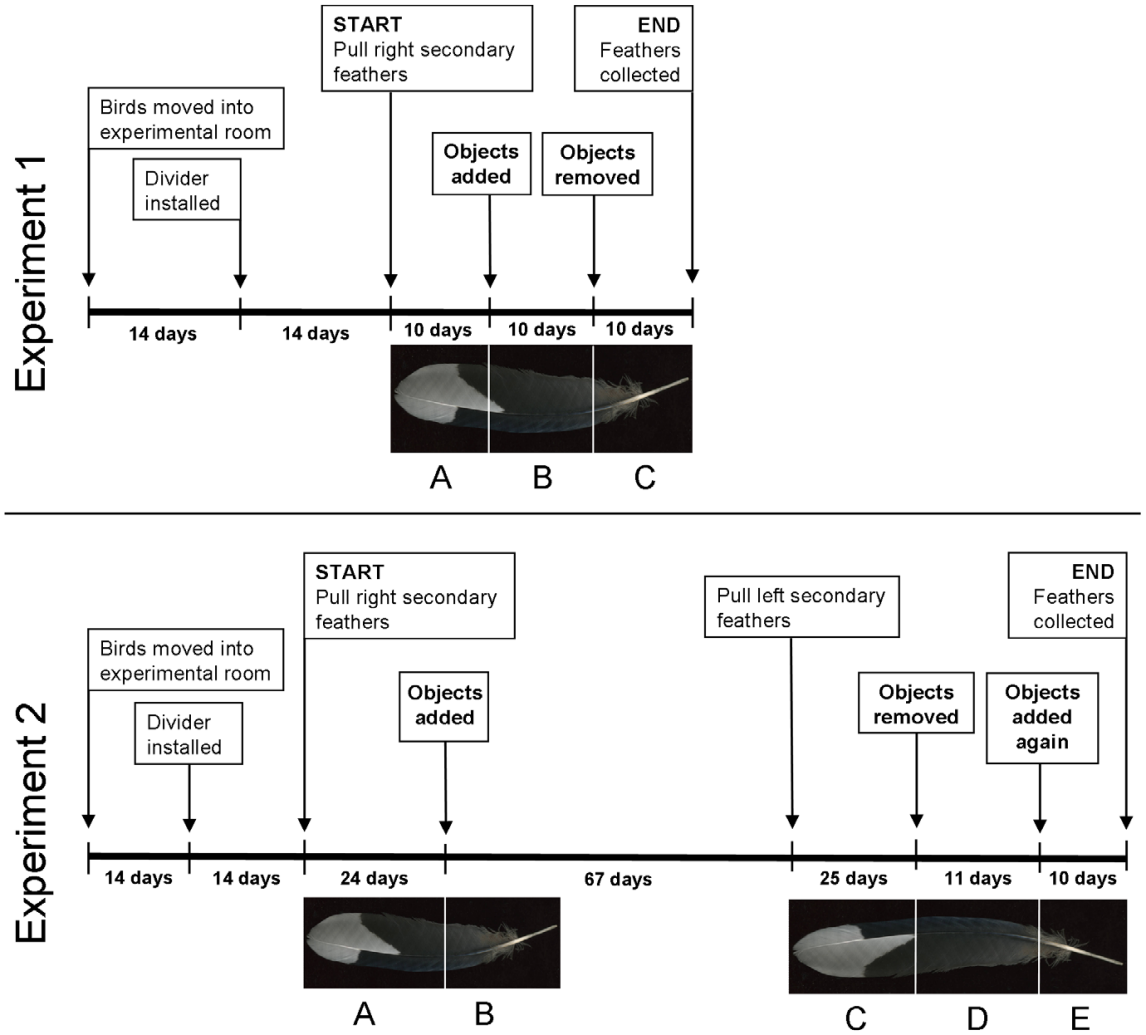
Experimental timelines with diagrams illustrating how feather sections reflect periods of the experiments. Feathers from experiment 1 were cut into three sections corresponding to periods prior to pre-enrichment (A), short-term enrichment (B), and removal of enrichment (C). The first feather from experiment 2 was cut into two sections corresponding to pre-enrichment (A) and short-term enrichment (B), and the second feather from experiment 2 was cut into three sections corresponding to long-term enrichment (C), removal of enrichment (D), and non-novel enrichment (E). See text for descriptions of time periods. Note: illustrations not to scale.

Beginning in October 2007, 16 randomly selected nutcrackers (8 male, 8 female) were moved from the colony room into a similar windowless experimental room and assigned randomly to one of two walls that faced each other. After 2 weeks, a plastic curtain was installed that divided the experimental room in half: eight birds on one side of the divider were visually isolated from eight birds on the other side of the divider. Birds were allowed to adjust to the divider for an additional 2 weeks (Fig. 1).

We then pulled the right secondary feather #1 (adjacent to primary #1) from each nutcracker to induce new feather growth. All feathers pulled were fully grown and dead. Subsequent feather growth was measured every 5 days for the remainder of the experiment. After the first 10 days of feather growth enrichment objects to which the birds were naïve were installed in the cages of birds on one side of the divider only, thus separating birds into an enriched experimental group (n = 8) and a non-enriched control group (n = 8). Enrichment objects comprised three plastic bird toys (balls: 195 mm×45 mm, rings: 190 mm×50 mm, and a mirror lantern: 140 mm×35 mm) and one wooden chew toy (250 mm×55 mm) that all hung inside the cages, plus an artificial pine garland (300 mm×300 mm) installed on the outside of the upper left back corner of the cage. All objects were added at the same time. After 10 days of enrichment, objects were removed and the regrowing feathers were allowed to grow for an additional 10 days, at which point they were pulled out (Fig. 1).

We chose to group all experimental birds together on one side of the room rather than randomly assign treatments to birds. Random assignment would have resulted in experimental birds being neighbors with non-experimental control birds and to reduce obvious bias we would have been forced to visually isolate neighbors. We chose to not do this for two reasons. First, it would have been difficult to keep control birds from seeing enrichment objects during cage changes. Second, and more importantly, little is known about nutcracker sociality, but it is suggested that they are moderately social birds [62]. Birds in our experiments had always been maintained in a colony setting, so rather than confound our data by subjecting birds to a potentially stressful social situation, we chose to group birds by treatment, thus allowing individuals within a treatment group to see each other.

### Experiment 2

Beginning in October 2008, 23 nutcrackers (12 m, 11 f) that had not previously received cage enrichment were selected randomly, moved into the experimental room, and assigned randomly to one of two walls that faced each other. The birds' daily routine was maintained throughout the experiment and was the same as that described above. We repeated the adjustment procedure as in experiment 1, except that the divider separated nutcrackers into groups of 11 and 12. We then pulled a feather from each bird to induce new feather growth (Fig. 1). All feathers pulled were fully grown and dead. We pulled the right secondary feather #1 from 17 of the 23 birds, but six birds were missing or growing that feather so we selected the next morphologically similar feather in sequence: right secondary feather #2 (4 birds) or #3 (1 bird), or right primary feather #1 (1 bird).

Regrowing feathers were measured every 5 days until feathers were approximately half-grown (mean ± SD  = 61.74±8.95 mm). Then, as in experiment 1, enrichment objects to which the birds were naïve were installed in the cages of birds on one side of the divider only, forming an enriched group (n = 11) and a non-enriched control group (n = 12). All objects were added at the same time. The induced feathers were allowed to complete their growth and were subsequently pulled 31 days later to ensure that feathers were fully grown (Fig. 1).

Once birds had been exposed to enrichment objects for 67 days, we pulled a second feather from each individual to induce new feather growth (Fig. 1). All feathers pulled were fully grown and dead. We pulled left secondary #1 from 17 birds, but three birds were missing or growing that feather. For those individuals, we selected the next morphologically similar feather in sequence: left secondary feather #4 (1 bird), or right secondary feather #1 (2 birds) or #2 (1 bird). Feathers were allowed to grow for 25 days, at which point the enrichment objects were removed from cages. After 11 days the enrichment objects were re-installed into the same cages as before and feathers were allowed to grow for a final 10 days before being collected for analysis. We were only able to sample second feathers from 21 birds because two birds died and, in a third, an induced feather did not regrow.

### Feather sections

We cut all feathers using growth measurements as guides such that cut sections corresponded to the different time periods of the experiment (Fig. 1). Feathers from experiment 1 were cut into three sections: the distal section was grown prior to enrichment (Fig. 1, top, A: “pre-enrichment”), the middle section was grown while enrichment objects were present in the cages (Fig. 1, top, B: “short-term enrichment”), and the proximal section was grown after objects were removed (Fig. 1, top, C: “removal of enrichment”). The first feathers from experiment 2 were cut into two sections: the distal section was grown prior to enrichment (Fig. 1, bottom, A: “pre-enrichment”) and the proximal section was grown while enrichment objects were present in the cages (Fig. 1, bottom, B: “short-term enrichment”). The second feathers from experiment 2 were cut into three sections: the distal section was grown while enrichment objects were still present in the cages (Fig. 1, bottom, C: “long-term enrichment”), the middle section was grown after objects were removed (Fig. 1, bottom, D: “removal of enrichment”), and the proximal section was grown when objects were re-installed (Fig. 1, bottom, E: “non-novel enrichment”).

### Behavior

We recorded nutcracker behavior during experiment 2 using a small digital video camera that was able to record all birds on one side of the room simultaneously (Table 1). The camcorder was mounted on a small tripod that stood on a utility cart that was used for daily feeding and was therefore familiar to the birds. We used the digital timestamp on the recordings for calculating elapsed time.

**Table 1:** Observation schedule for nutcracker behaviors during Experiment 2.

Experimental period	Time of day (hrs)	Behavior^1^		
		LTF	AL	EXP
Pre-enrichment	1700		**x**	**x**
Short-term enrichment	1100	**x**		
	1700		**x**	**x**
Long-term enrichment	1100	**x**		
	1700		**x**	**x**
Removal of enrichment	1100	**x**		
	1700		x	x

Observation schedule for nutcracker behaviors recorded during Experiment 2. LTF = latency to feed, AL = activity level, EXP = exploration. See text for definitions of behaviors.

1 All behaviors were measured for 5 mins.

We measured latency to feed (LTF) for each nutcracker as the time taken by a bird to approach its food dish after being reintroduced into the cage. Previous studies have used latency to feed as a measure of fearfulness [13], [63]–[65]. Our nutcrackers normally feed readily in the presence of caretakers, and even jump on food cups and begin to feed before the cups are fully snapped into place on the cage. Thus, we knew *a priori* that the birds should not be afraid of caretakers or food during feeding. We made 5-minute video recordings of both experimental and control nutcrackers during normal feeding time (∼11:00 hrs) on three separate occasions as they were released into fresh cages that already had food in cups. The first recording was made immediately following the installation of enrichment objects into experimental cages. This recording captured the initial behavioral responses of all birds at the time when experimental individuals were first exposed to enrichment objects. The second and third recordings were made when birds had received 2 months of continuous exposure to objects (“long-term enrichment”), and immediately following removal of enrichment objects 26 days later (“removal of enrichment”).

Birds are known to express behaviors ranging from freezing to active investigation when exposed to novelty [64]. We therefore measured two behaviors likely to vary with exposure to novel objects: activity level (AL), quantified by counts of all hops around the cage and positional changes (i.e., turning around 180° but remaining in the same place when perched); and exploration (EXP), quantified by counting the number of pecks at any object within the cage or at any part of the cage itself. Recordings of AL and EXP lasted 5 minutes, as in previous work (e.g., [31]), and were made several hours after afternoon water changes (at ∼1700 hrs) and in the absence of caretakers to ensure that these behaviors were not affected by human presence. Observations of AL and EXP were made during four periods of our experiment: 2 weeks prior to enrichment (“pre-enrichment”), immediately following the installation of enrichment objects (“short-term enrichment”), after birds had 2 months of continuous exposure to objects (“long-term enrichment”), and immediately following removal of enrichment objects 26 days later (“removal of enrichment”).

### Feather CORT assays

We extracted CORT from feathers in three separate extractions using a methanol-based technique following [60]. We measured the lengths of all feathers, and then cut, removed, and discarded the calamus. The remaining feather sample was cut with scissors into very small pieces (<5 mm^2^) and 10 mL of methanol (HPLC grade, VWR International, Mississauga, Ontario, Canada) was added. We placed samples in a sonicating water bath at room temperature for 30 min, then incubated them at 50°C overnight in a water bath. We separated the methanol from the feather material by vacuum filtration, and the methanol extract was placed in a 50°C water bath and allowed to evaporate in a fume hood. Extracts were later reconstituted in a small volume of phosphate buffer system (PBS; 0.05 M, pH 7.6) and frozen at −20°C until analyzed by radioimmunoassay (RIA). We assessed the efficiency of each of the three methanol extractions by including feather samples spiked with a small amount (approximately 5000 CPM) of ^3^H-corticosterone in each extraction (see Appendix S1 in [60] for more details). On average, greater than 95% of the radioactivity was recoverable in the reconstituted samples.

Feather CORT levels were determined by RIA as in previous studies [60], [61], [66]–[68]. Measurements were performed on reconstituted methanol extracts and were duplicated. Samples were measured in four assays with an intra-assay coefficient of variation of 7.4%, an inter-assay coefficient of variation of 14.1%, and mean (± SD) limit of detection (ED80) of 12.9±2.2 pg CORT/assay tube. Data values are expressed as pg CORT per mm of feather, which gives a valid estimate of CORT per unit time of feather growth (see [60], [61], [69] for validation). CORT assays were performed at the University of Saskatchewan, Canada.

### Statistical analyses

To determine if feather sections used for CORT analyses differed in length between enriched and non-enriched controls we combined all data from both experiments and used a mixed model (PROC MIXED; SAS v. 9.1). We used length of feather section as the response variable, treatment as the explanatory variable, and experiment (i.e., 1 or 2) as a random factor. We included a repeated statement to account for multiple measurements taken from the same individual over time [70].

We used mixed modeling (PROC MIXED) to compare CORT values from feather sections grown during the periods of our experiments. For experiment 1 we compared pre-, short-term, and removal of enrichment. For experiment 2 we compared pre-, short-term, and long-term enrichment; removal of enrichment; and non-novel enrichment. We modeled feather CORT as the response variable, time period and treatment (i.e., experimental or control) as fixed factors, and included a time period × treatment interaction term. We used a repeated statement to account for multiple measurements taken from the same individual over time. Two feathers were collected from each bird in experiment 2, so our model included a random factor to account for possible variation between feathers.

To determine the influence of enrichment on behavior, and to address the relationship between behavior and CORT, we used mixed models (PROC MIXED and PROC GLIMMIX; SAS v. 9.1). We modeled behaviors individually, using behavioral data (LTF or AL) as the response variable, feather CORT as a covariate, and time period, treatment (i.e., control or experimental), and a time period × treatment interaction term as fixed factors. LTF data were fitted to models using a normal error distribution and an identity link function in PROC MIXED. AL data were counts and were therefore fitted to models using a negative binomial error distribution and a log link function in PROC GLIMMIX. We used random statements to account for variation between feathers, and used repeated (PROC MIXED) or random (PROC GLIMMIX) statements to account for the multiple measurements taken from the same individual over time.

EXP data were counts, but were zero-inflated and we were not able to get models to converge using PROC MIXED or PROC GLIMMIX. Instead, we used a zero-inflated Poisson model (PROC GENMOD) to address the relationship between EXP and CORT. We used counts of pecks as the response variable, CORT as a covariate, and time period and treatment (i.e., enriched or control) as explanatory variables. Although this approach had the advantage of accounting for the high incidences of zeros in our data, it did not allow us to include an interaction term or random or repeated statements. However, considering how few counts of pecks were actually recorded throughout the experiment, we do not believe the absence of interaction and random terms affected our results significantly.

## Results

Lengths of feather sections used for CORT analyses did not differ between control and experimental groups in any time period of either experiment (*F*_1,142_ = 0.09, *p* = 0.77).

### Experiment 1

**Figure 2:**
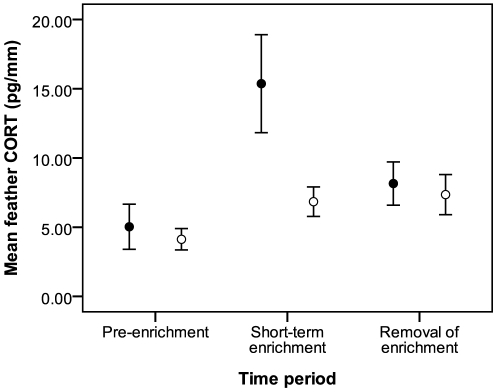
Mean (± SE) nutcracker feather CORT values (pg/mm) from experiment 1. Filled circles: experimental birds; open circles: non-enriched controls. See text for explanation of time periods.

Overall, mean CORT values differed significantly between time periods (*F*_2,22_ = 13.76, *p*<0.001; Fig. 2), but not between treatment and control birds (*F*_1,12_ = 3.96, *p* = 0.07), and the time period × treatment interaction was not significant in our model (*F*_2,22_ = 2.76, *p* = 0.09). However, *post hoc* comparisons revealed that CORT levels increased significantly from pre-enrichment to short-term enrichment in experimental birds (*t*_1,22_ = −4.66, *p*<0.001), but not in controls (*t*_1,22_ = −1.57, *p* = 0.13). CORT values from the enrichment removal period did not differ significantly from pre- enrichment values for either group (experimental: *t*_1,22_ = −1.47, *p* = 0.16; control: *t*_1,22_ = −1.92, *p* = 0.07).

### Experiment 2

**Figure 3:**
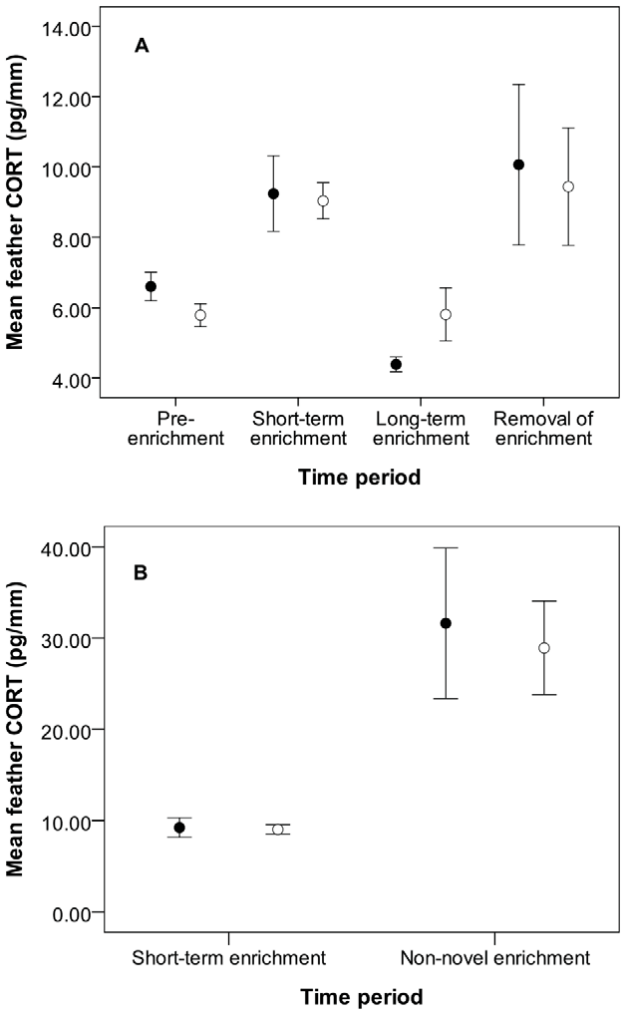
Mean (± SE) nutcracker feather CORT values (pg/mm) from experiment 2. Filled circles: experimental birds; open circles: non-enriched controls. (A) shows the first four time periods of the experiment; (B) shows the fifth time period (non-novel enrichment) and includes short-term (novel) enrichment for comparison. Note different scales on y-axes. See text for explanation of time periods.

Similar to experiment 1, mean CORT values differed significantly between time periods (*F*_4,93_ = 16.48; *p*<0.0001; Fig. 3), but not between treatment and control (*F*_1,93_ = 0.07; *p* = 0.79), and there was no significant interaction between treatment and time period (*F*_4,93_ = 1.92, *p* = 0.20). CORT increased significantly between pre-enrichment and short-term enrichment periods for both experimental (*t*_1,93_ = −3.82, *p* = 0.0002) and control birds (*t*_1,93_ = −4.71, *p*<0.0001). Long-term enrichment CORT values in controls were similar to pre-enrichment values (*t*_1,93_ = 0.03, *p* = 0.98), but in experimental birds long-term enrichment CORT values were significantly lower than pre-enrichment values (*t*_1,93_ = 3.16, *p* = 0.002). CORT levels from feather sections grown after removal of enrichment objects increased significantly from long-term enrichment levels in experimental birds (*t*_1,93_ = −2.63, *p*<0.01) but not in controls (*t*_1,93_ = −1.86, *p* = 0.07). CORT levels increased significantly following non-novel enrichment for both control and experimental birds and were significantly higher than both pre-enrichment (*t*_1,93_ = −5.15, *p*<0.0001) and enrichment levels (*t*_1,93_ = −4.49, *p*<0.0001).

### Behavior

**Figure 4:**
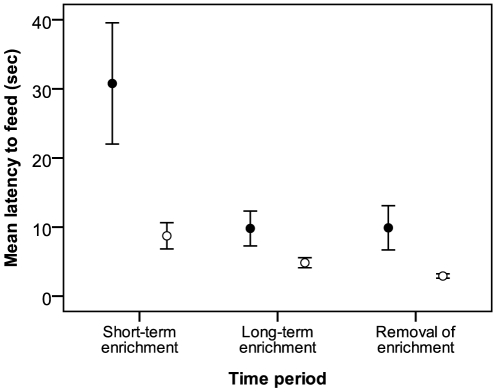
Mean (± SE) latency (sec) to feed (LTF) of nutcrackers during experiment 2. Filled circles: experimental birds; open circles: non-enriched controls. See text for explanation of time periods.

There was a significant interaction between the effects of treatment and time period on LTF (*F*_2,52_ = 4.30; *p*<0.02). During short-term enrichment, LTF was significantly greater in experimental birds than in controls (t_1,52_ = −2.58; *p* = 0.01; Fig. 4), but this effect disappeared during long-term enrichment when LTF in experimental birds was reduced (t_1,52_ = 3.89; *p* = 0.0003) to levels seen in controls. CORT was not significantly related to LTF in either group (*F*_1,52_ = 1.33; *p* = 0.25).

**Figure 5:**
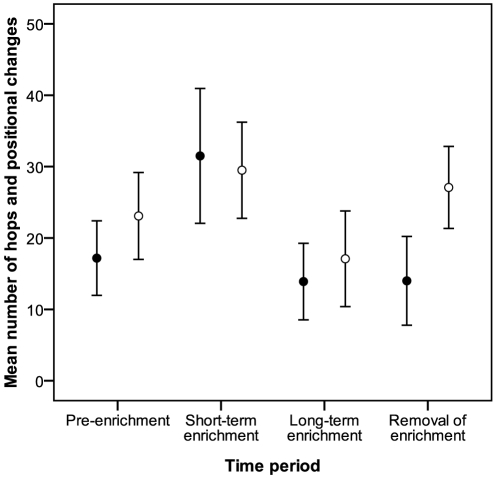
Mean (± SE) counts of nutcracker hops and positional changes (activity level, AL) during experiment 2. Filled circles: experimental birds; open circles: non-enriched controls. See text for explanation of time periods.

There was no significant interaction between treatment and time period (*F*_1,73_ = 0.11; *p* = 0.96) in our model of AL, so we interpreted the main effects directly. AL did not differ between time periods (*F*_1,73_ = 0.88; *p* = 0.45; Fig. 5) or between treatments (*F*_1,73_ = 0.72; *p* = 0.40), and was not significantly related to CORT (*F*_1,73_ = 0.50; *p* = 0.48).

**Figure 6:**
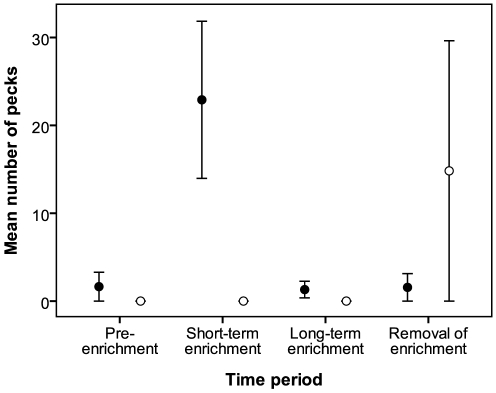
Mean (± SE) counts of nutcracker pecks (exploration, EXP) during experiment 2. Filled circles: experimental birds; open circles: non-enriched controls. See text for explanation of time periods.

Our model of EXP revealed that, overall, experimental birds were more likely to show pecking behavior than control birds (Wald chi-square = 5.5; *p*<0.01; Fig. 6) and that pecking was significantly more likely during the short-term enrichment than in other periods (Wald chi-square = 13.33; *p*<0.001). EXP was not related to CORT (Wald chi-square = 0.26; *p* = 0.61) for either group.

## Discussion

Did enrichment attenuate stress physiology in captive nutcrackers? The integrated measure of CORT increased significantly from pre-enrichment levels following short-term exposure to enrichment objects in two separate experiments, indicating that stress physiology was likely enhanced, not reduced, following the manipulation. However, individuals exposed to long-term enrichment (i.e., the final 25 days of a 92-d enrichment period) expressed CORT levels that were significantly lower than pre-enrichment values, suggesting a physiological benefit. These results led us to hypothesize that nutcrackers perceived initial enrichment as a stressful change of environment, and we predicted that the change of environment associated with removal of enrichment would likewise produce elevated CORT levels. Contrary to our predictions, CORT levels in experiment 1 decreased following removal of enrichment and returned to pre-enrichment levels. However, in experiment 2, CORT levels increased significantly to levels seen following short-term enrichment, in accordance with our predictions.

How do we explain these seemingly opposite responses to removal of enrichment in our two experiments? The main difference between experiments 1 and 2 was length of exposure to enrichment objects prior to removal. The longer exposure likely allowed birds in experiment 2 to habituate to the objects such that the objects became part of their normal environment, similar to acclimation seen in previous studies ([71], [72] and see [24]). The fact that CORT levels during long-term enrichment (the final 25 days of a 92-d enrichment) were not significantly higher than those prior to enrichment provide evidence that birds had indeed adjusted to the presence of objects. Thus, removal of the objects in experiment 2 constituted a change of environment to which birds responded by elevating CORT. This was not the case in experiment 1 where the shorter 10-day exposure period did not allow for the same length of adjustment. Objects were therefore not recognized as part of the environment and instead were perceived as a stressor. Thus, the removal of the objects was seen as a removal of a stressor, to which birds responded by decreasing CORT secretion, albeit non-significantly, to pre-enrichment levels.

Were the birds physiologically stressed by short-term enrichment? Although none of the birds showed any sign of illness or discomfort during either of our experiments, two birds died during, and one shortly after, experiment 2. Interestingly, all three fatalities were in the experimental group, and those individuals had three of the four highest CORT values for the short-term enrichment period. The provisioning of enrichment must be done carefully and in the context of the species and environment. It is possible that the installation of four objects inside the cage was perceived as an over-enrichment by nutcrackers, and the birds reacted with sustained CORT responses. Sustained elevated CORT secretion can suppress immune response [23], [73], so elevated CORT levels may have been a contributing factor in the deaths. However, CORT values during short-term enrichment, when responses would be expected to be most robust, were not the highest seen in the experiment, so we doubt that birds were over-enriched. Thus, we cannot conclude that enrichment *per se* caused stress sufficient enough to result in these deaths.

Behaviorally, only LTF and EXP behavior were influenced by the addition of enrichment objects, but these responses were not seen two months later, nor were they seen upon removal of the objects. By contrast, feather CORT increased significantly in periods following both the addition and removal of enrichment objects. Importantly, a lack of relationship between feather CORT levels and concurrently measured behaviors support the idea that stress-related behavior and GC physiology may act independently of each other within an individual, and highlights a physiological response that was not detectable through the behaviors we measured. Despite evoking a physiological response to enrichment, is possible that the objects were not engaging enough to elicit anything other than transient behavioral responses. This is in contrast to previous studies that found behavioural, but not physiological, responses to enrichment [19], [20], [39], [40], [43]. These results suggest that both context and type of enrichment are important determinants of responses to enrichment.

Control birds showed similar feather CORT levels to experimental birds in all but one period of experiment 2, despite not being able to see enrichment objects. Although we made every effort to ensure that control birds were treated identically to experimental birds other than not receiving enrichment objects, we cannot rule out the possibility that some factor common to both groups influenced our results. Controls were only visually, and not aurally, isolated from their experimental counterparts. Nutcrackers are semi-social corvids that possess complex vocal communication systems [62]. Nutcrackers in both treatment groups were noisy throughout the experiment, so it is possible that vocalizations from experimental birds were changed or interrupted by the presence of enrichment objects, and the perception of these altered vocalizations promoted a release of CORT by control birds. Public information can affect CORT levels in birds [74], so it is possible that vocalizations work in a similar way, alerting individuals. This intriguing possibility requires further research.

Our results challenge the notion that all types of enrichment are immediately beneficial for captive animals and indicate that initial reactions to enrichment need not be positive or, in the case of some behaviors, may even be absent. Furthermore, a lack of relationship between feather CORT and the behaviors we measured suggests that relying solely upon behavioral measures of stress to assess captive animal well-being can be misleading, an assertion supported by other studies [19], [20], [39], [40], [43]. We are not suggesting that enrichment is harmful to captive animals. In fact, our results indicate that long-term enrichment generally reduced activity of the HPA axis and therefore may provide some physiological relief for captive animals experiencing otherwise stressful conditions. This result was not seen in control birds, so the effect was likely caused by exposure to enrichment objects. Furthermore, the fearfulness we observed in response to short-term enrichment was not detected following long-term enrichment, suggesting that this effect was temporary.

Likewise, although we did not detect a behavioral response to removal of enrichment objects, we are not suggesting that impoverishment has no behavioral consequences. On the contrary, numerous studies have documented deleterious behavioral effects of impoverished environments (e.g., [39], [40]). However, in our study we were only concerned with behavioral responses occurring very soon after experimental manipulations; as we did not detect any change in the behaviors we measured following removal of enrichment objects, we conclude that nutcrackers did not respond behaviorally to removal of enrichment.

Previous work has suggested that novelty is an important property of enrichment to which animals respond [13], [16], [65], but results have been mixed [75]. Our experimental design allowed us to assess the effect of novelty on nutcracker GC secretion because birds in experiment 2 were re-enriched with objects to which they had previously been exposed. Had novelty been a factor influencing GC responses in the short-term enrichment period in experiment 2, feather CORT values during the non-novel enrichment period (i.e., second exposure to the objects) should have been lower than or equal to levels following initial exposure. To the contrary, we found feather CORT values to be significantly higher when birds were no longer naïve to the enrichment objects. This indicates that nutcrackers mounted a stronger physiological response the second time they were exposed to the same objects, a result previously seen only in mammals [75]. The non-novel enrichment period in our study was only 10 days, compared to 25 days for the short-term enrichment period, so it is plausible that the shorter growth period resulted in a higher average CORT. However, the feather sections in experiment 1 were also grown over 10 days, yet their CORT values were half that of the non-novel enrichment values in experiment 2 and comparable to the values from longer feather sections grown during experiment 2. Thus, it appears that the length of feather growth period is not responsible for the high CORT values seen during the non-novel enrichment period. As an alternative explanation, the experimental birds may have developed a negative association between the enrichment objects and the activities of our experiment. Although all nutcrackers in our experiment had been handled daily for several years prior to our experiment, the extra handling required for measuring feather lengths and collecting feathers may have been perceived as stressors by the birds. The enrichment objects may thus have acted as a cue and upon seeing the enrichment objects a second time birds responded more strongly because they had a negative prior experience.

### Conclusions

Enrichment can undoubtedly alter both physiological and behavioral functioning in animals [32], [35], [38], [45], [76], [77]. By demonstrating that an integrated measure of GC physiology varies significantly with changes to enrichment in the absence of agonistic interactions, our study sheds light on potential mechanisms driving those physiological and behavioral effects. Our work adds an avian perspective to studies addressing GC responses to novelty, and suggests that when a non-novel stimulus acts as a cue, acclimation may be overridden by negative past experience. Importantly, our findings suggest that how animals perceive enrichment and its removal depends on the duration of exposure: shorter-term enrichment may be experienced as a stressor, but longer-term enrichment allows for acclimation and therefore subsequent removal of enrichment constitutes a change to the environment. Future research should work to identify the factors that affect the rate at which individuals transition between these two psycho-physiological states. Studying such factors in captive and free-living animals will improve our understanding of how and why animals adapt to environmental change.
