# Correction: Does Environmental Enrichment Reduce Stress? An Integrated Measure of Corticosterone from Feathers Provides a Novel Perspective

**DOI:** 10.1371/annotation/3ac615cc-2ecf-4d3e-9281-3b9b9b04cf08

**Published:** 2011-03-17

**Authors:** Graham D. Fairhurst, Matthew D. Frey, James F. Reichert, Izabela Szelest, Debbie M. Kelly, Gary R. Bortolotti

There was an error in the author contributions section. The corrected author list for the category "Conceived and designed the experiments" is: "GDF GRB JFR IS DMK."

